# Supplementary material for: Global and Arctic climate sensitivity enhanced by changes in North Pacific heat flux
Source: Nat Commun. 2018 Aug 7;9:3124. doi: 10.1038/s41467-018-05337-8 (PMC6081422; doi:10.1038/s41467-018-05337-8)
Supplement: Supplementary file 1 — Supplementary Information [file 41467_2018_5337_MOESM1_ESM.pdf]

Supplementary Information for:
Global and Arctic climate sensitivity enhanced by changes in North Pacific heat flux
by Praetorius et al.

Supplementary Figures

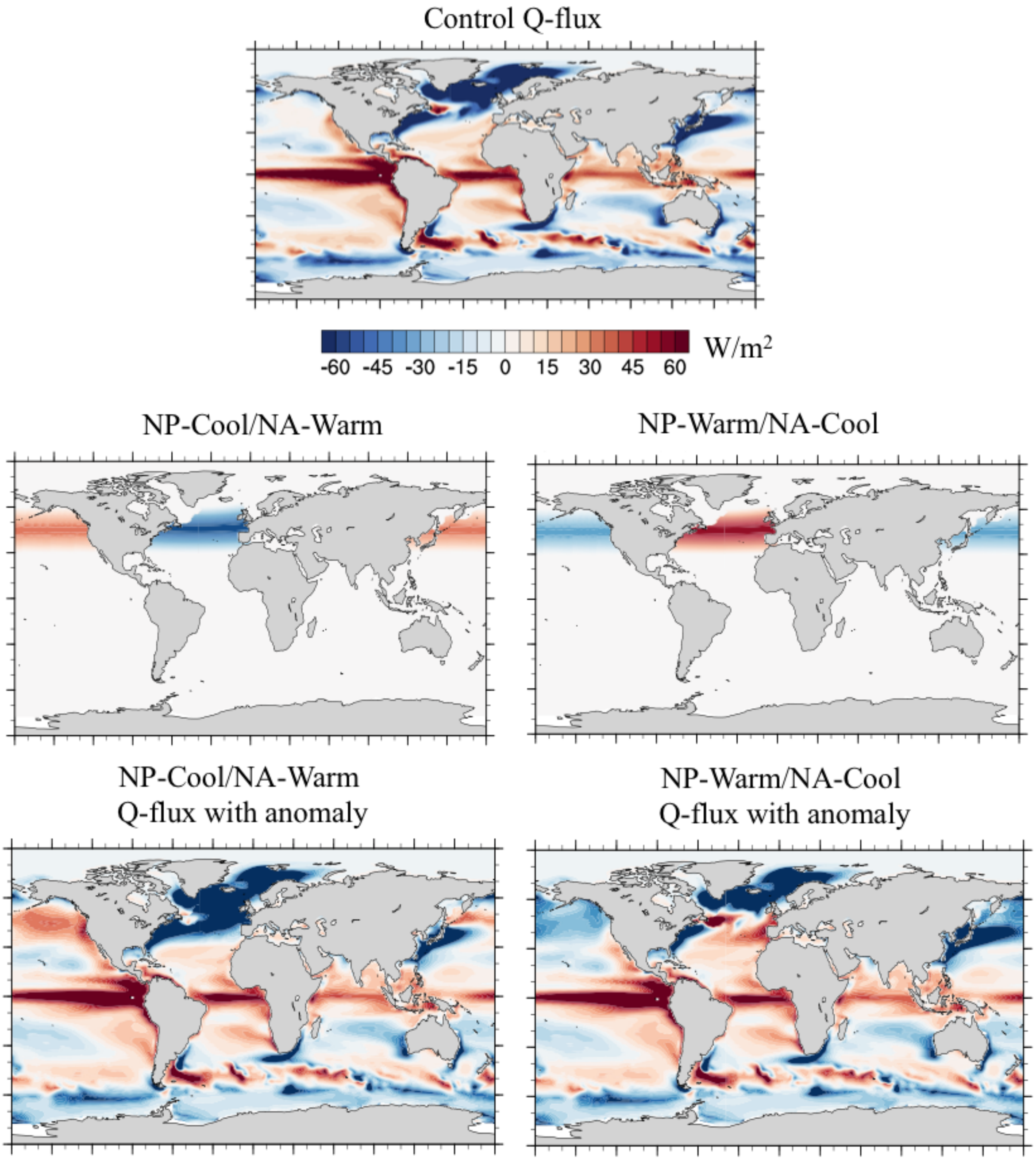

Supplementary Figure 1. Heat flux (Q-flux) modifications employed in the seesaw simulations. The annual average control Q-flux (top), the imposed Q-flux anomalies for the seesaw experiments (with a global Q-flux perturbation summing to zero) (middle row), and the resulting annual average Q-flux with the anomalies (bottom row). The same spatial pattern of Q-flux modification is applied for each of the single basin simulations, only without compensating heat flux in the adjacent basin. A positive heat flux is here defined as going from the atmosphere into the ocean and a negative heat flux as coming out of the ocean into the atmosphere.

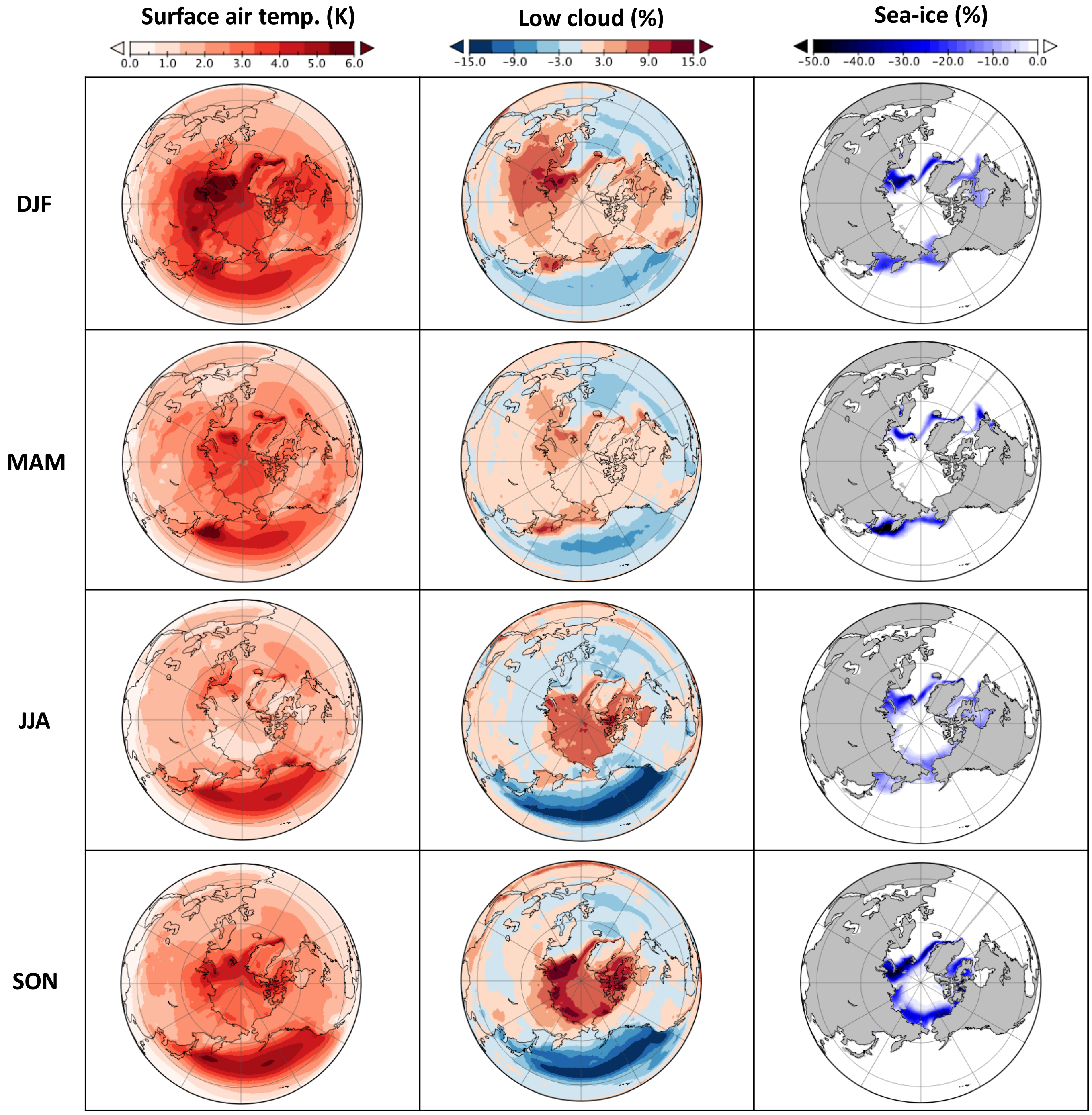

Supplementary Figure 2. Surface air temperature, vertically-integrated low cloud area, and sea-ice concentration anomalies in the NP-Warm simulation for December – February (DJF), March – May (MAM), July – August (JJA), and September – November (SON).

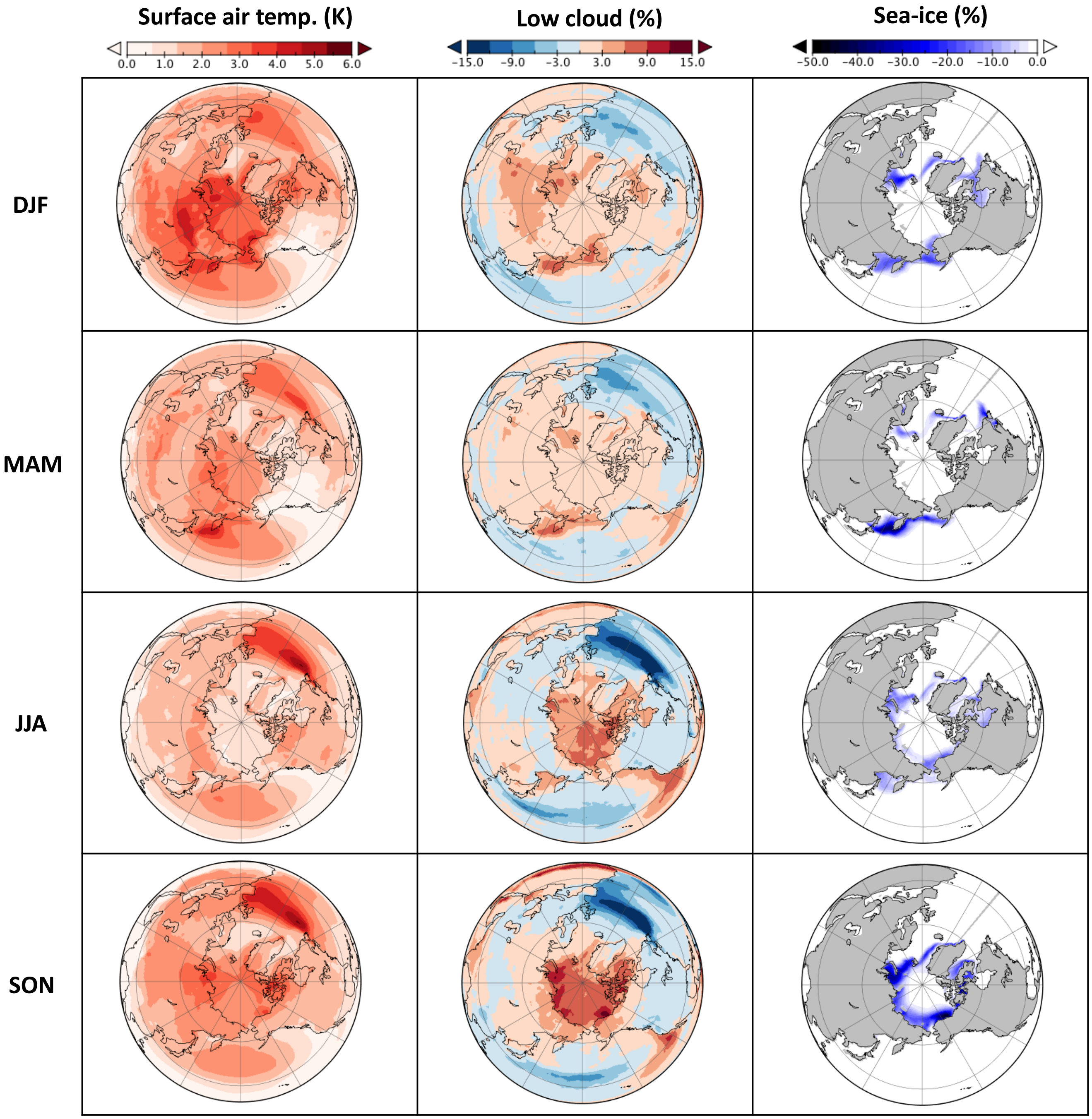

Supplementary Figure 3. Surface air temperature, vertically-integrated low cloud area, and sea-ice concentration anomalies in the NA-Warm simulation for December – February (DJF), March – May (MAM), July – August (JJA), and September – November (SON).

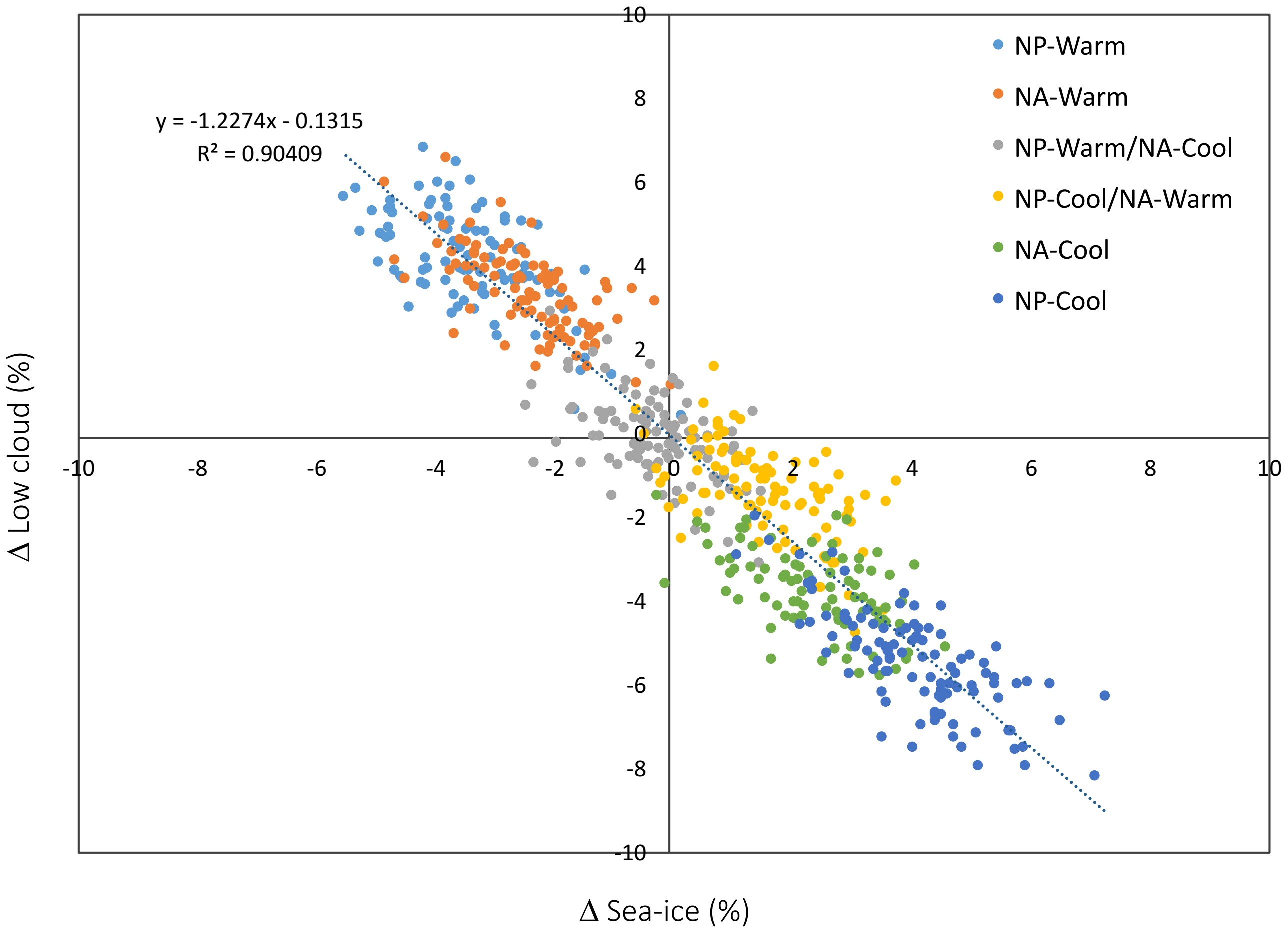

Supplementary Figure 4. Linear regression between the annual change in sea-ice area and vertically-integrated low cloud area in the various simulations for the Arctic region.

A

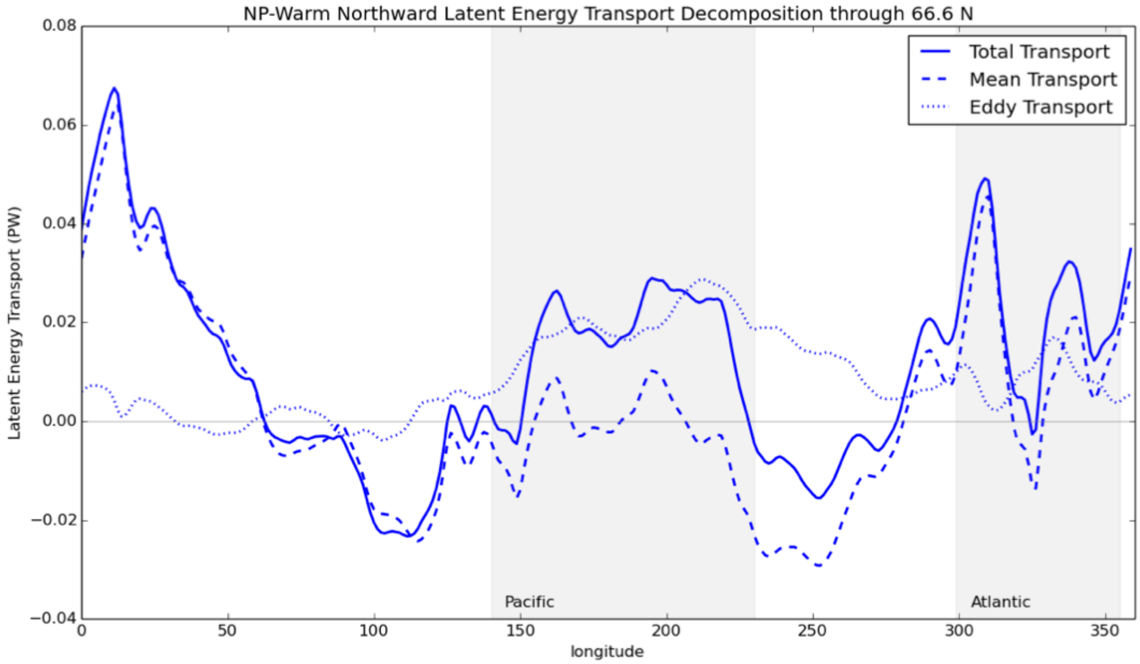

B

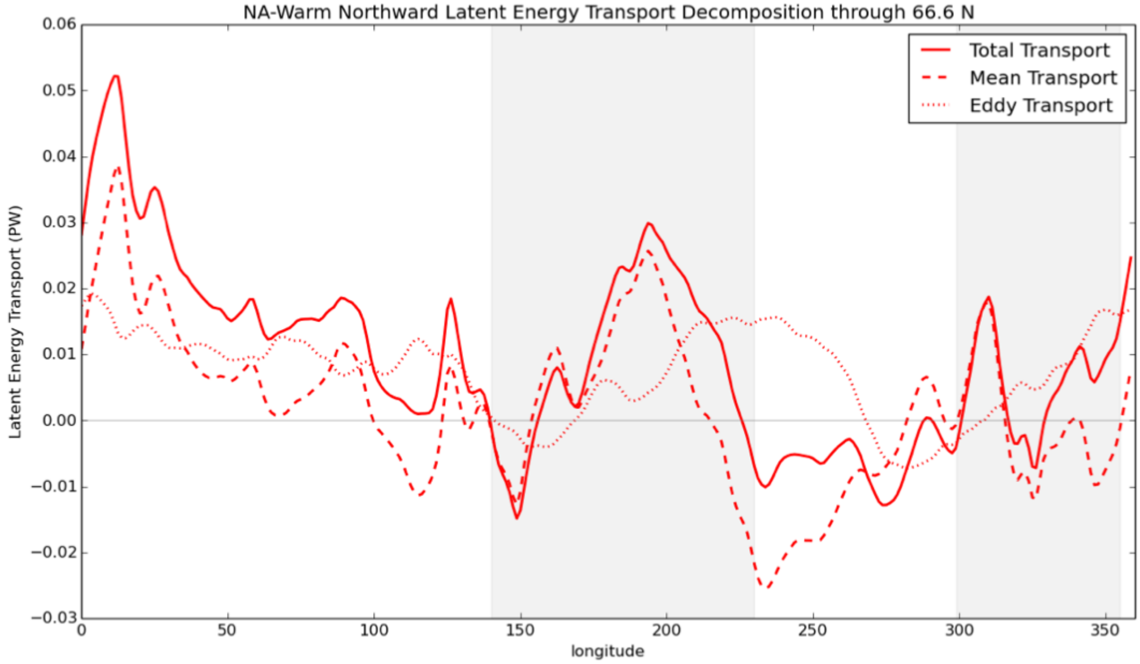

Supplementary Figure 5. Decomposition of the zonal distribution of the mean and eddy components of the total northward latent energy transport across 66.6°N in the NP-Warm simulation (A) and NA-Warm simulation (B). Shaded regions denote the Pacific and Atlantic basins.

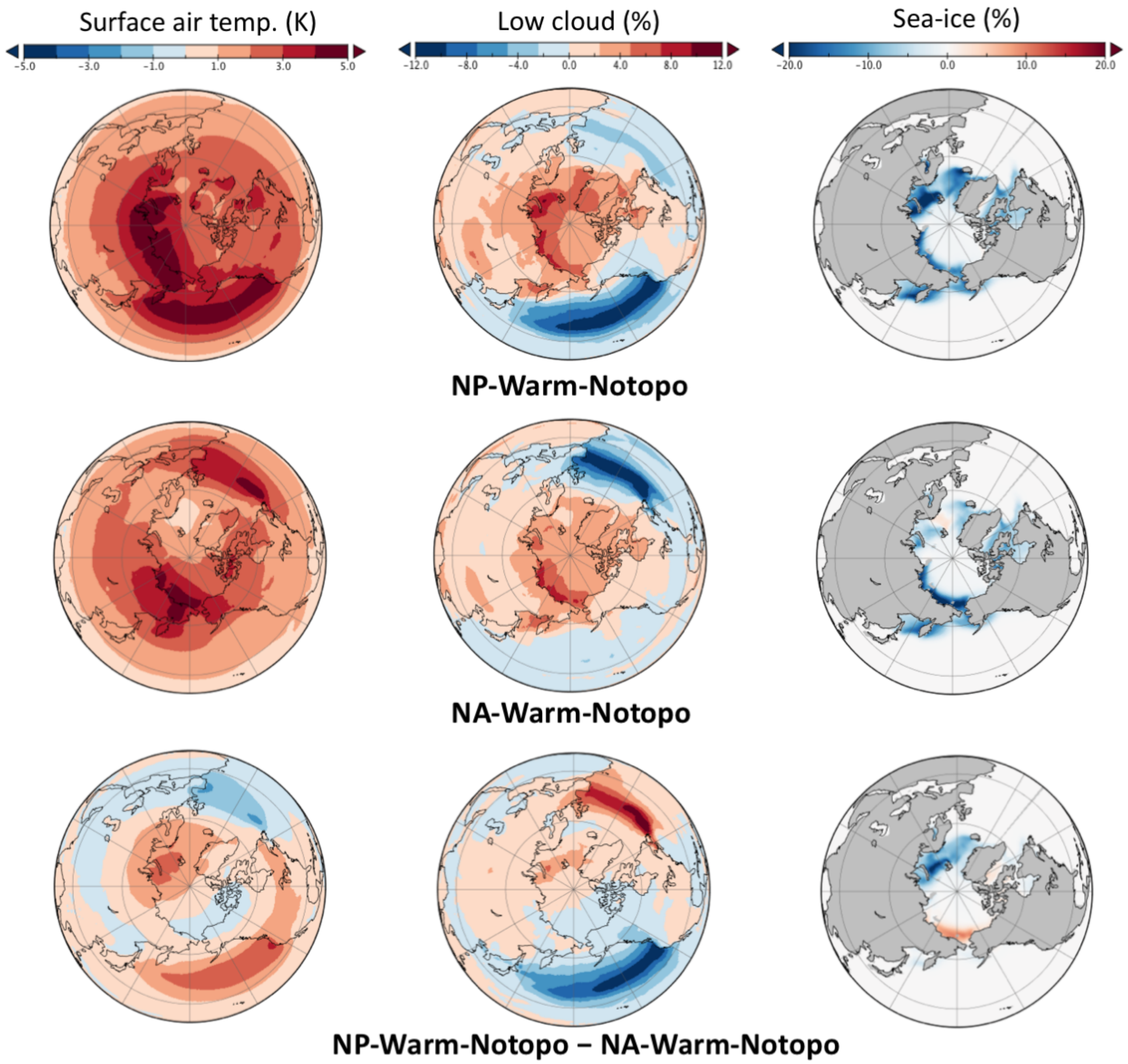

Supplementary Figure 6. Annual surface air temperature, vertically-integrated low cloud area, and sea-ice concentration anomalies in the positive North Pacific and North Atlantic heat flux simulations with land topography set to sea level. NP-Warm-Notopo (top), NA-Warm-Notopo (middle), and the difference between NP-Warm-Notopo and NA-Warm-Notopo (bottom).

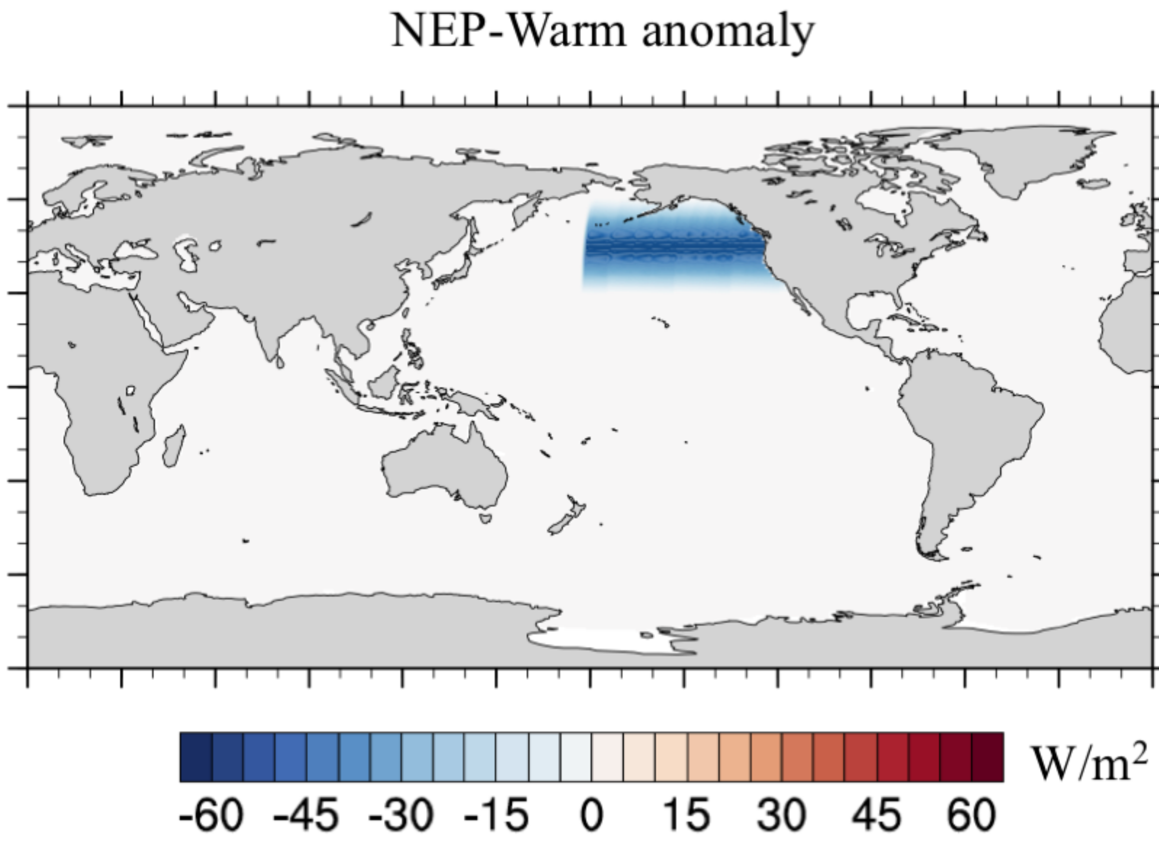

Supplementary Figure 7. Modified North Pacific heat flux to be constrained in an area equivalent to the North Atlantic heat flux perturbation (NEP-Warm).

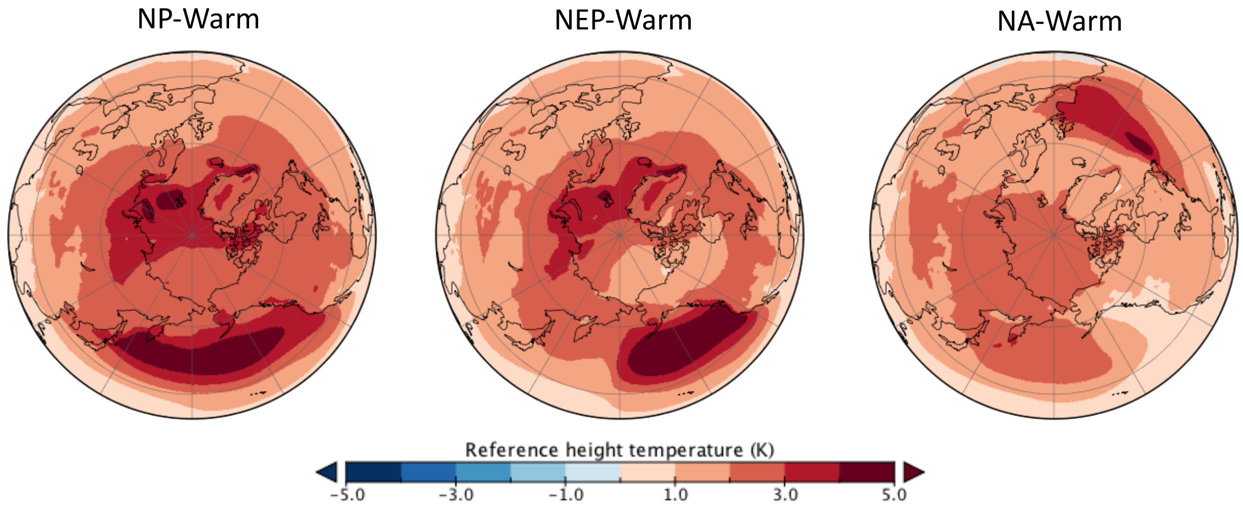

Supplementary Figure 8. Comparison of surface air temperature anomalies for the NP-Warm, NEP-Warm, and NA-Warm simulations. All simulations are the global equivalent of + 1 W/m^2 forcing from the ocean to atmosphere. The NP-Warm heat flux anomaly is more diffusely spread across the entire Pacific basin between 30-60°N, whereas the NEP-Warm heat flux anomaly is constrained to the Northeastern Pacific in an area equivalent to the NA-Warm heat flux anomaly.

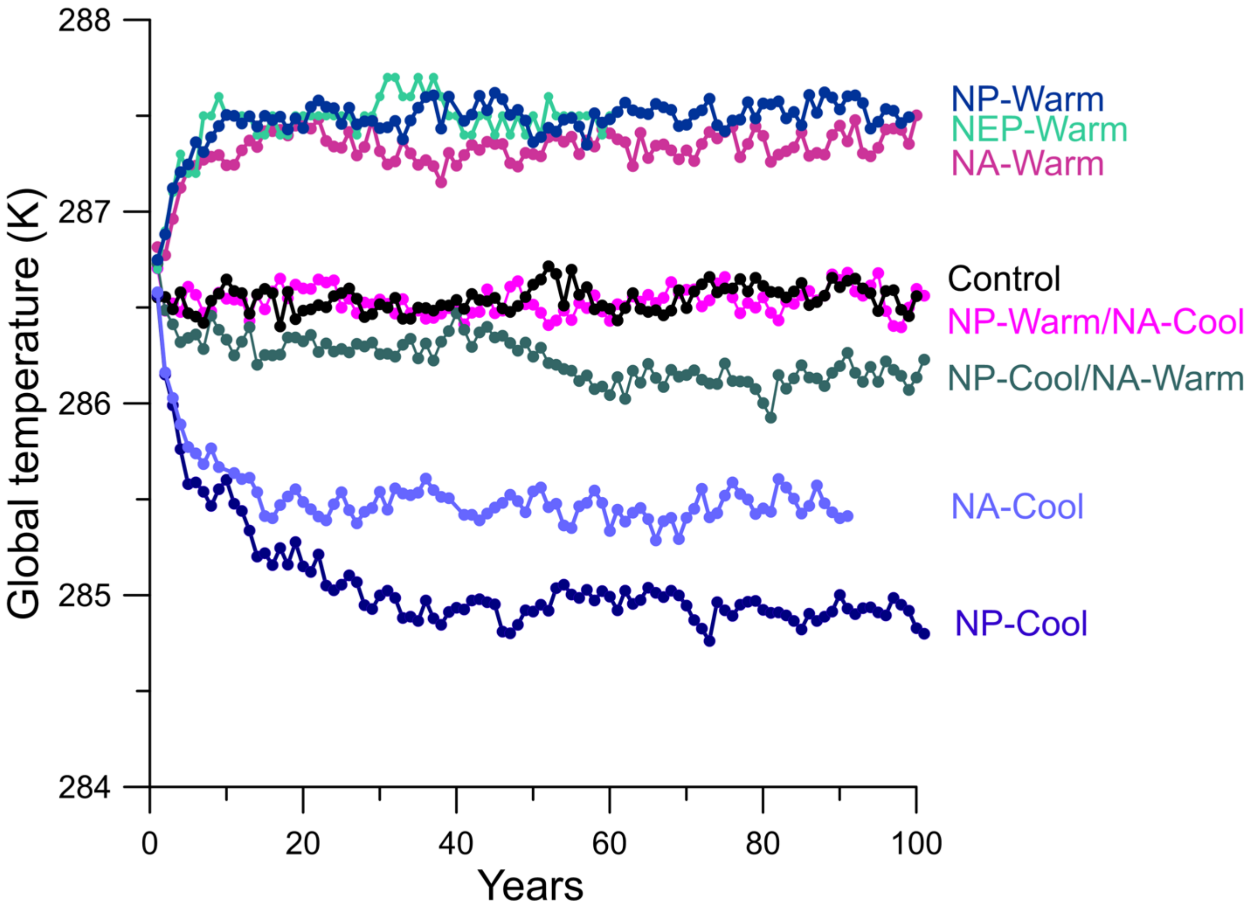

Supplementary Figure 9. Annual mean global surface air temperatures in the various simulations.

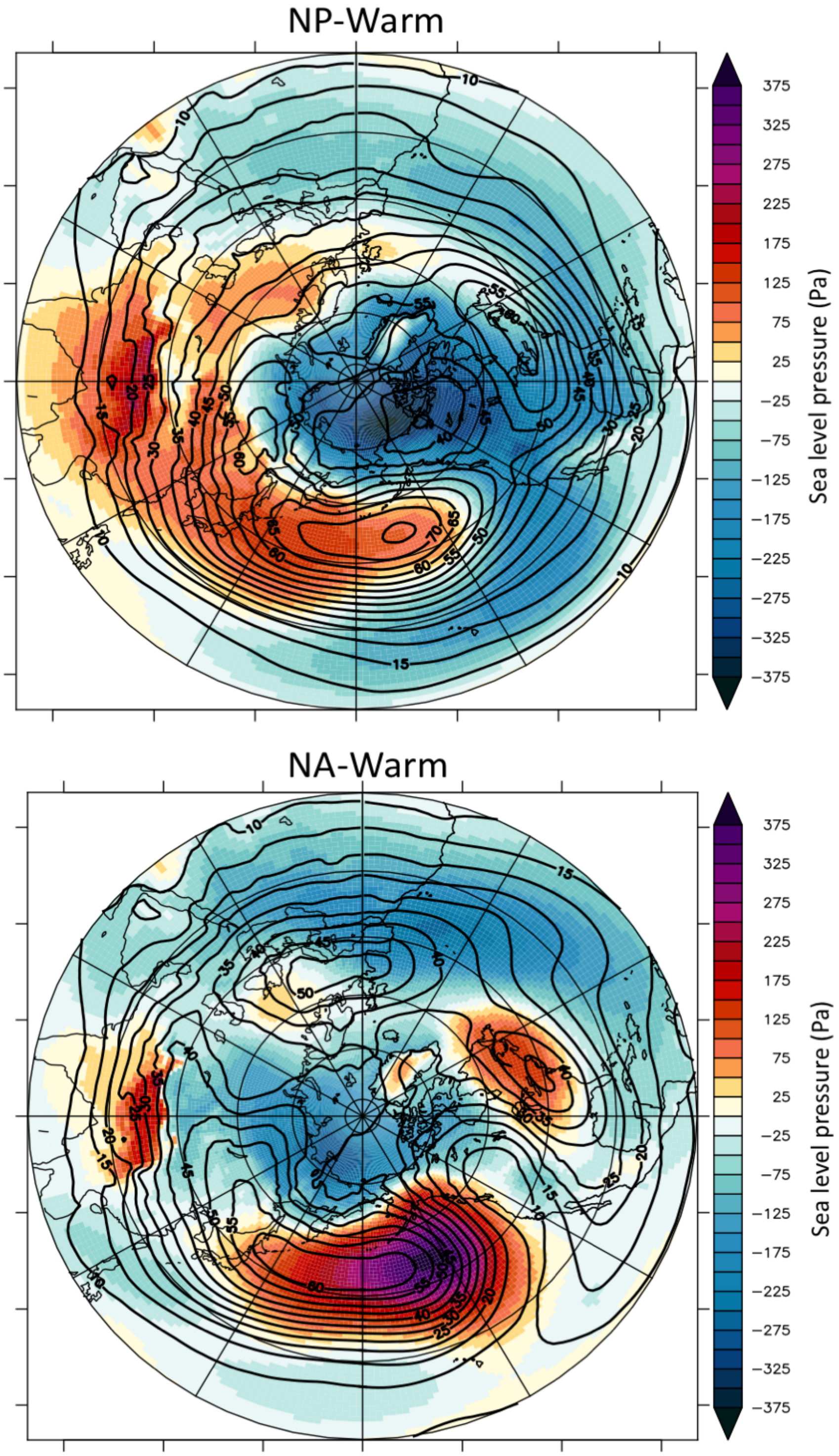

Supplementary Figure 10. Surface pressure anomalies in the NP-Warm and NA-Warm simulation during winter (Dec-Feb) (color shading) with the 500 mb height pressure anomalies overlaid in contours. The NA-Warm simulation exhibits a high-pressure anomaly centered over the North Pacific, signaling a weakening of the winter-time Aleutian Low.

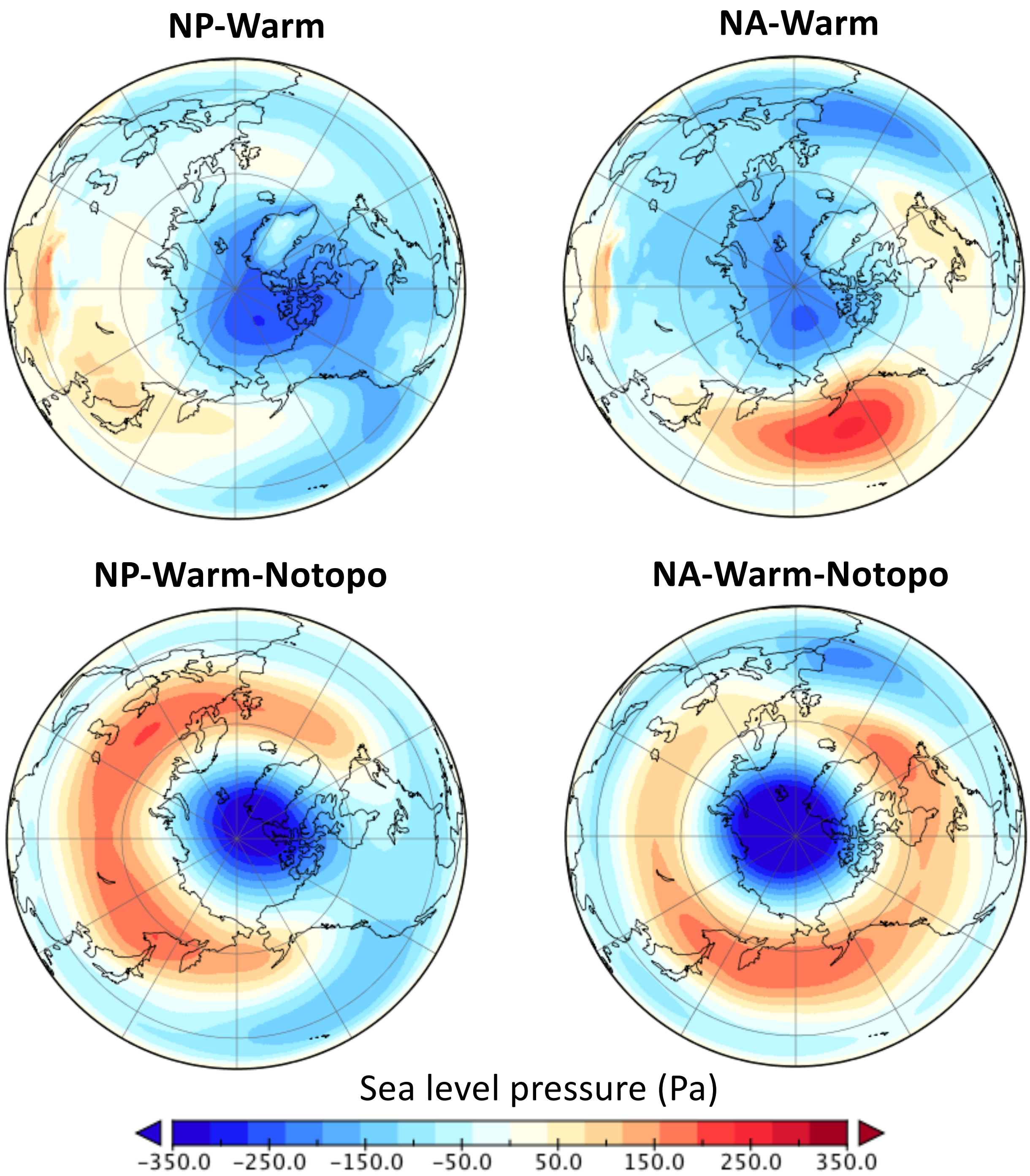

Supplementary Figure 11. Annual sea-level pressure anomalies in the NP-Warm, NA-Warm, NP-Warm-Notopo, and NA-Warm-Notopo simulations. Both the NA-Warm and NA-Warm-Notopo sea-level pressure anomalies result in high pressure anomalies over the central North Pacific and North American continent. In contrast, the NP-Warm and NP-Warm-Notopo result in a low pressure 'breach' centered on North America.

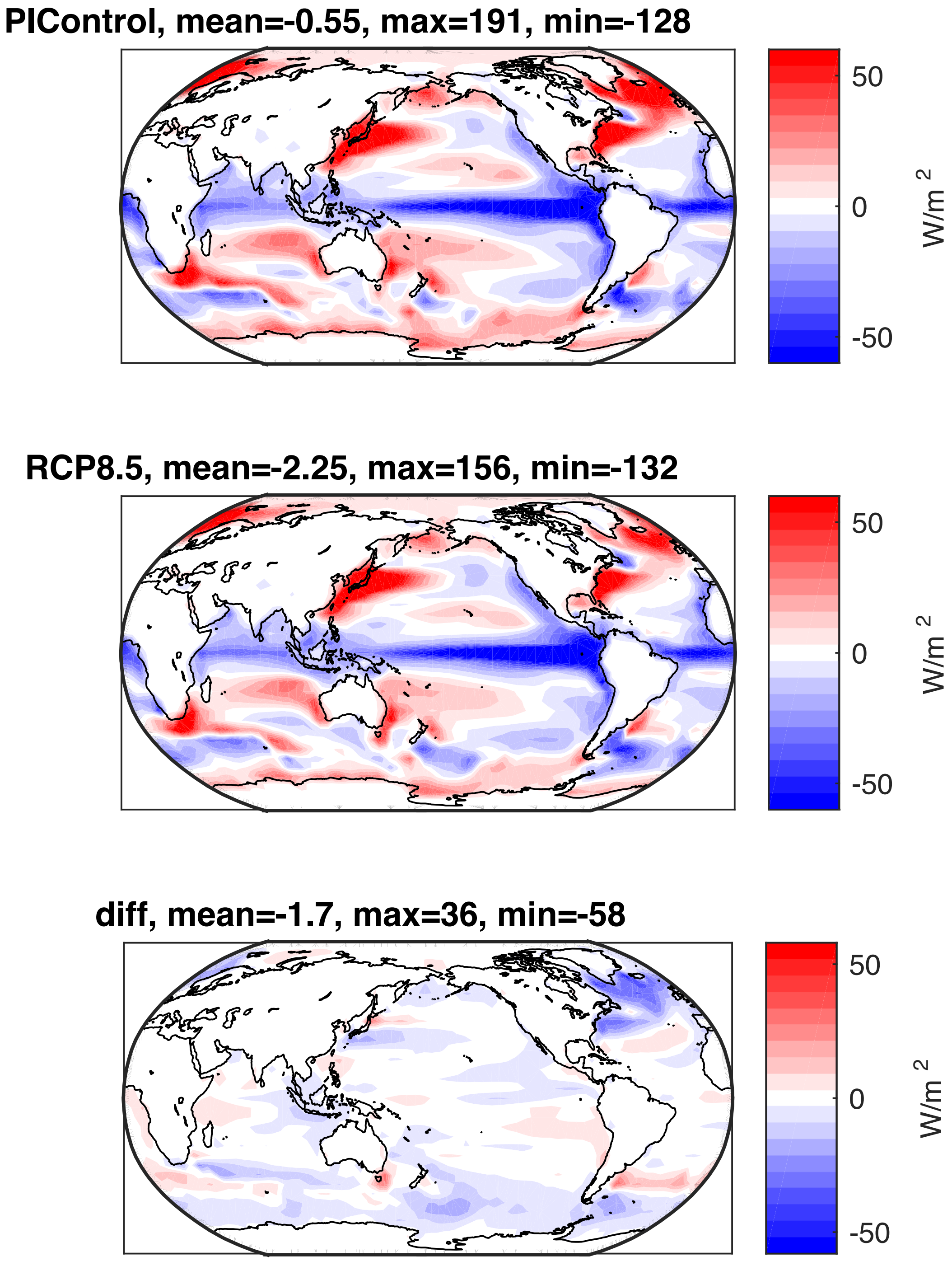

Supplementary Figure 12. Annual averaged surface heat flux in CMIP5 for the preindustrial control (top panel; average for years 0-200), the RCP8.5 scenario (middle panel; average for years 2081-2100), and the RCP8.5 anomaly relative to the preindustrial control (bottom panel). In this case, surface heat flux is defined as positive from the ocean to atmosphere.
